# Exploration of gynaecological cancer high dose-rate brachytherapy treatment: a pilot study

**DOI:** 10.11604/pamj.2018.30.27.14608

**Published:** 2018-05-15

**Authors:** Alicia Ehlers, Chandra Rekha Makanjee

**Affiliations:** 1 Steve Biko Academic Hospital, Department of Oncology, Pretoria, South-Africa; 2 University of Pretoria, Department of Radiography, South-Africa; 3 Department of Medical Radiation Sciences, Faculty of Health, University of Canberra, Bruce, ACT, Australia, 2617

**Keywords:** Gynaecology, cancer, expectations, experiences, understandings, high dose-rate brachytherapy treatment

## Abstract

**Introduction:**

Performing brachytherapy on female patients with gynaecological cancer is a sensitive procedure, apart from it being an intricate investigation. The aim of this study: establishing gynaecological cancer patients' expectations, experiences and understandings of the high dose-rate brachytherapy treatment procedure. Exploring these patients' life-worlds provides further insights into improving their preparedness and experiences.

**Methods:**

A qualitative research design with a descriptive phenomenological research approach was used. Recruitment entailed using purposive sampling. To obtain rich insights into the lived experiences, data was acquired through semi-structured interviews until data saturation.

**Results:**

Major challenges were experienced with mixed thoughts and feelings, from negative trauma to desires being fulfilled. These patients should have received more information regarding their upcoming brachytherapy treatment. This would have alleviated some of their fears and anxieties, which would in turn have provided them with a better brachytherapy treatment experience. Envisaged outcomes and desires are used to cope. Patients influence each other negatively, so they rather wanted to “see for themselves”.

**Conclusion:**

Despite some disappointments and negative experiences throughout the brachytherapy continuum, the patients were left with a positive outlook, saying that it is an excellent and necessary treatment. Further studies should be done to elaborate on fulfilments of recommendations in the brachytherapy department of radiation oncology.

## Introduction

The exploration on the experiences of gynaecological cancer patients' undergoing brachytherapy was found to be of a difficult nature [1-3]. Cancer patients are often fearful and experience anxiety [4, 5] when going to the radiotherapy department because of a lack of knowledge of, and/or misconceptions about the treatment [6, 7]. It is acknowledged that being informed does not alleviate all of the patients' fears, but rather contributes to the preparation of patients on what to expect and how to cope when undergoing a radiation related treatment procedure, for instance brachytherapy [4, 7-9]. Thus, the aim of the study was to explore, by means of using a qualitative research approach instead of the traditional survey method, [10,11] to get insights into patients' expectations, experiences and understandings prior to, whilst undergoing and post- high dose-rate (HDR) brachytherapy treatment processes and procedures. Gynaecological cancer presents with 500 000 cases annually, worldwide, of which the mortality rate is about 50% [12]. Gynaecological cancers are the second most widespread cancer, worldwide, among women. In Southern Africa, it is found that gynaecological cancer is the most common in black women [13,14]. It is estimated that 83% of all gynaecological cancer cases occur in developing countries [13,14]. Gynaecological cancer thus needs specific attention due to the vulnerability of the female cancer patient group and the sensitivity of the treatment. Amongst other treatment strategies, gynaecological cancer patients are also referred to the oncology department for treatment [15]. Should the patient be referred for treatment(s) it may involve external beam radiation therapy (EBRT), brachytherapy and/or chemotherapy whereby a radical approach is taken, [15] with brachytherapy being an invasive procedure [15].

The treatment procedure entails the delivery of EBRT by means of a teletherapy unit (for example a linear accelerator), accompanied by intra-cavitary brachytherapy [15]. The brachytherapy treatment used in the local public hospital's Department of Oncology entails the use of a HDR remote after-loader. Applicators are inserted into the vaginal vault (close to the cervix) of the patient by the oncologist [15]. The radio-active source can then move from the lead housing, through the applicators, irradiating the cervix (and uterus if necessary) from a close range [15]. A higher dose of radiation is then delivered to the cervix and involved area than to the adjacent normal tissue [15]. At the beginning of each treatment procedure the patient receives sedation and analgesia in this department and all other institutions [16]. It is applied differently or in different amounts, in various departments, but has the same goal, namely to alleviate the patient's pain and help her to relax [16]. The patient undergoes this brachytherapy treatment process approximately three to four times, with each treatment being conducted in a normal time-span of 20 to 30 minutes (during the time-frame when the data was gathered, at the specific source decay time), as time differs according to the half-life of the source [15,16]. The number and dosages of treatments are dependent on the type/stage of cancer [15]. High-quality patient care is of utmost importance [17, 18], especially because of brachytherapy being such a sensitive and invasive procedure. The health system is there to serve the people and responsiveness plays a significant role in patient-centred care [17, 18]. This includes meeting patients' expectations, respect for patients and their wishes, and optimal communication between health workers and patients [17, 18]. The interactions and communication are very important in brachytherapy as they play a significant role in the patient's experiences of the “what” and “how” of the brachytherapy as part of their treatment journey [6].

## Methods

A qualitative research design was used by applying a descriptive phenomenological research approach [19]. Thick description was obtained of the participants' expectations, experiences and understandings of the brachytherapy treatment processes and procedure to be captured [19]. The researcher followed the process of intuiting by looking at the phenomenon and to capture the “lived experience” of the patient before, during and after the HDR brachytherapy treatment processes and procedures [19]. This study was conducted in a radiation oncology department at an urban tertiary hospital's brachytherapy unit, situated in the Gauteng region. To ensure privacy and confidentiality, all patient interviews were conducted in a designated location. A research interview room was used, which was located away from possible interference whilst the interview was conducted. The population and sampling were determined by entries in the register of patients undergoing radiation treatment in conjunction with brachytherapy. Consent forms were devised and explained to the patient who consented prior to the commencement of the treatment procedure. Purposive sampling was utilised to obtain rich information from the participants [19]. Patients of diverse cultures were included in this study to obtain a holistic patient spectrum. Only ten participants were interviewed as data saturation was achieved [20]. The name of every patient was kept confidential; instead, each patient was assigned a code. The semi-structured interview schedules were guided by D Long's approach on cervical cancer brachytherapy patients' treatment experience [16]. The structure of the interview was amended to include probes for expectations and understandings, also further shaped by the participants' responses: i) The initial interview schedule: this was conducted prior to the patients' first HDR brachytherapy treatment procedure to explore their expectations and anticipated experiences. The first question was: Why are you here today? Then probes were added. ii) The post treatment interview schedule: this was done directly on completion of the first treatment was completed to establish the patients' experiences of the HDR brachytherapy treatment they had undergone. The question: what made you decide to give consent for the brachytherapy treatment? was changed to: how did you come to know of this treatment? iii) The exit interview schedule: this was done following completion of all the HDR brachytherapy treatments. The purpose of this interview was to establish whether their expectations and experiences had altered and/or presented an opportunity to share new experiences and to verify the participants' previous expectations shared and understandings and knowledge obtained during the process.

They were also asked: how do you think we could improve on making your experience better' The interviews were audio recorded and transcribed as per verbatim, with each interview lasting about 30 minutes. The participants' “life world” is described, as developed by Giorgi (1985-2003). Giorgi delineated four essential steps of analysis: A) Reading for a sense of the whole; B) Differentiating the description into meaning units; C) Reflecting on the psychological significance of each meaning unit; D) Clarifying the psychological structure of the phenomenon [21]. The following steps were undertaken: an overall impression and identifying themes were gained by dividing the text into meanings units [22]; meanings were then condensed across cases; and generalising descriptions given for each theme, forming the foundation for new descriptions and concepts [22]. The quality and trustworthiness of the study was ensured by using these strategies by applying rich and thick description. The aim of the data collected was to give enough information on the results to the reader to facilitate an evaluation on the credibility of the results. A paper trail was kept by the researcher throughout the research study [23]. Member checking was done to ensure credibility and accuracy of the data captured and the interpretation thereof [24]. The cyclic process was applied, as described by Kemmis: plan → act → observe → reflect (and then again plan → etc) [25, 26]. Going through this cycle aids in responsiveness, this in turn assists in obtaining rigour. The early cycles help to determine the content of the later cycles, and then in the later cycles the interpretations can be tested, challenged and refined [26]. Each cycle consists of critical reflection; the researcher recollects and critiques the occurrences in each step [26].

**Ethical approval**: The Research Ethics Committee of the University of Pretoria and Faculty of Health Sciences Ethics committee approved this study. All participating institutions granted permission for the study and all potential patient participants signed written informed consent before enrolment in the study.

## Results

The approach of the interpretation and analysis was underpinned by the “lived experience” [27] of the participants, as they journeyed through the brachytherapy processes and procedures. This was from an individual experience, as well as the various points of contact and/or interactions with the healthcare providers who directly or indirectly mediated through the various occurrences.

**“I'll see for myself”**: When gynaecological cancer patients were asked about their reason for being at the hospital on that current day, they immediately acknowledged and referred to their illness: *“Because I am sick, I've got the cancer,” (Pt1PreB:12); “It's because I am sick, they've discovered that I've got cancer,” (Pt4PreB:12-14); “Want ek is siek, ek het die cancer,” (Because I am sick, I have the cancer) (Pt5PreB:13)*. Their illness status and the EBRT were mostly familiar to them at this stage: *“I'm getting treatment for the cancer. I'm getting the radiation treatment,”* (Pt1PreB:12). There was still an uncertainty about brachytherapy to most: *“I don't know, they just took me that side, they say I must come this side, I don't know… I don't know what is happening this side,” (Pt4PreB:22-24)*. Patient participants preferred not to be influenced by fellow patients. They wanted to experience the HDR brachytherapy treatment first hand: *“When the other patients want to tell me anything about this treatment, I say no, you mustn't tell me anything, I'll see by myself. They told me, oohh, you will get sick from that treatment, and it is sore if they treat you. But I say, “no”, don't tell me, I will see for myself when I'm at that treatment. Because the people, they want to scare some people. So, I just tell them, that uhhhm, “no, stop it”*.

**“The machine was hot, but this one is not hot”**: External beam radiotherapy treats with photons, externally, whereas brachytherapy treats with a radio-isotope, internally [15]. Participants' experiences of the actual procedure were varied and they described it according to some past experiences of what they were familiar with: *“It's fine, it was little bit different than the other treatment, but it is fine. They put it like this,” (showing to pelvis). The machine was hot, but this one is not hot, (Pt3PostB:5, 8)“*. Overall, the patients had a positive outlook on the brachytherapy treatment: *“Okay, it went nicely, the Brachy worked nicely to me,” (Pt1Tele:11-12*). But the pain was excruciating for some: *“I experienced a lot of pain, so very sore. Both treatments were very sore, but the second one, luckily was a bit quicker, that helped. The first one almost killed me. I was almost dead,” (Pt5ExitB:5; 12-13)*. These patient participants presented with the ability to endure and tolerate the pain involved because of the hope the treatment provides them.

**“Now I understand that brachytherapy”**: The patient participants interpreted the procedure they had undergone and the side-effects thereof by constructing their own meaning. *“They put that machine under the womb and then it hurts. They want to see this disease, how much there is,” (Pt6PostB:14)*. Others described it as some form of cleansing: *“They push my womb so and then they clean.” (Pt2PreB:17-18), or like, “cleaning the dead cells” (Pt7PostB:37*). The procedure was also defined by a physical illustration-that is, with a hand movement on the pelvis when explaining their understanding: *“They put it like this,” (showing to pelvis) (Pt3PostB:6)*. The position of the equipment was mentioned and its effect: *“I experienced they put the machine here, it's not that bad,”* (Showing to the pelvic area) (Pt6Exit:16-17). The term “understand” was used when describing the brachytherapy procedure: *“Now I understand that that brachytherapy, work hard here,”* (showing to pelvic area) (Pt8Exit:17). Although some participants had experienced some form of difficulty during the treatment processes and procedures, they demonstrated contentment: *“No, that treatment is fine, I'm just happy. It's all a good condition. I thank you too much,” (Pt3Exit:21-22)*. A neutral stance was taken: *“No, nothing. I'm happy, I just want to be healed. Everything that you have done to me is fine,” (Pt4Exit:24-25)*. The term “brachytherapy” was commonly used amongst the cancer patients, whereas another found that patients rather referred to the “inside radiation” and experienced it as an operation [16]. Patients in this study described the brachytherapy as: “cleaning the dead cells” (Pt7PostB:37), *“cleaning the womb” (Pt2PreB:17-18), “some instrument inserted in my vagina” (Pt4PostB8), or “the doctor put that thing inside” (Pt8PostB:11-12)*.

**Patients' desires**: Most participants shared their desire to be healed and carry on living as normal people: *“I want to be healed. I want to be cured,” (Pt4PreB:26)*. However, some were concerned: *“I'm just a bit stressed that there is still some tumour left, I really hoped to be healed, I really wish it will go away,” (Pt7Tele:23-25)*. Some had doubts regarding the resurfacing of their illness and worries remained with some: *“I don't know if it (the cancer) can start again, that's the problem,” (Pt8PreB:17-18); whereas a few resorted to holding onto their faith: “I just say; God help me with it;” “I just hold onto my faith in God, God know what happened and God always love His children,” (Pt6PostB:7; 28-29)*. Women hope for dialogue with the nursing staff, even conversations unrelated to the treatment, these dialogues seem to help them find meaning and hope for the future [28]. In the current study, the nursing staff were only mentioned once by one patient. The patients seemed reluctant to talk to the doctors of their own accord, they were hesitant to seem too “pushy” or in coming across as too fearful in the face of “no” real danger [29]. Intimate questions/thoughts were instead rather shared with the researcher: “Can I have the drink” (Pt3Exit:26); *“Can I still have sex” and Can I still get the children” (Pt3Exit:14-15)*. Patients made suggestions on how the brachytherapy treatment processes and procedures could be improved, as related throughout the research document. The following conclusions were reached [16]: A) Informed consent: provide easily understood information in the home language of the patients'; B) Treatment related information: information sessions prior to treatment. Give patients a second explanation of the treatment procedure inside the treatment room; C) Informative material: provide patients with pamphlets/booklets on the disease and brachytherapy treatment procedure and possible side-effects; D) Impressions: show patients the brachytherapy treatment room and apparatus prior to the treatment; E) Feelings and concerns: utilise the time spent in the waiting room to prepare the patients psychologically for their forthcoming treatment; F) Coping strategies: prevent patients from being scared and stressed by informing them that they will be given a sedative before treatment delivery that will help them to not experience pain during treatment delivery; G) Treatment effects: administer adequate sedation medication. Administer a bigger dose of sedative (if necessary) for better pain control. Complete the treatment as quick and sufficient as possible.

## Discussion

Patients want the information they receive to be of positive value and tend to avoid too detailed or “unsafe” information [30]. Most participants avoided each other's negative comments and rather opted to see for themselves. Patients generally have has a need for valid and positive information, but the need only includes basic information regarding diagnosis, treatment options and common side-effects of radiation treatment. However, each patient's timing of desire for information varies, as does the level of detail and context [9, 30]. In a study done by Lesley Long on radiotherapy patients' informational needs, it was found that patients show a need for more information, and cancer patients prefer pamphlets/booklets [9]. It was found that some of the patients favoured verbal information in their vernacular or preferred language. Informing patients in a way they preferred could reduce anxiety and be used as a coping strategy [4, 5]. Stewart et al [31] found that in contradiction to Leydon [30]; women prefer to receive information from their physician or healthcare providers, and secondly, they also want printed information [30, 31]. The unfamiliarity with brachytherapy treatment will cause patients to be most anxious. As treatment progresses to succeeding treatments, the patient's fears will be somewhat subdued due to the familiarity and knowledge of what to expect regarding the procedure [32]. Healthcare providers act as a support structure to the patient, a sense of comfort. Hope is an intricate, multidimensional and active and prevailing factor in healing, coping, and quality of life [33]. Hope's relationship to caring and healing is evident in Cutcliffe's definition: “Hope is a multi-dimensional, dynamic empowering state of being, that is central to life, related to external help and caring, oriented towards the future and highly personalised to each individual” [34].

Even though there are different types of radiation treatment, including brachytherapy, they are seen as a source of hope. Although they fluctuated between hope and hopelessness [33], overall, these patients perceived brachytherapy as a beneficial treatment in terms of their illness. Brachytherapy, an invasive procedure, is a painful undergoing. Findings obtained from other literature were similar -pain levels experienced by patients varied between extremely painful and low to moderate pain [16]. Some patients were traumatised by the pain, whilst others were tolerant and not affected by it [16]. All participants mentioned some form of pain when asked about their brachytherapy experience. It varied from, *“sometimes it hurt” (Pt6PostB:4, 11)*, to *“I felt sore,” (Pt8PostB:5); “She had the pain,” (Translator; Pt3PostB:12)*, to *“Was very painful” (Pt4Exit:7); “Brachy is very sore,” (Pt7Exit:7*), and *“It was so painful,” (Pt9Exit:20)*. In a rectal brachytherapy study it was found that the patients, who were mostly males, who experienced the lithotomy position for the instrumentation aspect similar to this current study for the cervical cancer was very traumatising [35]. Women linked their experiences to previous familiar experiences, such as the pain of giving birth, or undergoing a pap smear [35]. Whereas the males felt embarrassed and exposed and found the lithotomy treatment position disempowering, intrusive and left them feeling vulnerable [35]. These authors found that preoperative HDR brachytherapy educational hand-outs that clarified the nature of the procedure helped the patients, both male and female, to be less anxious [35]. These patients have to live with the possibility that their cancer might return. Some patients hold onto their faith, whatever they went/go through, and as stated by Ellison, “Religious and spiritual beliefs and practices may improve the quality of a person's life by buffering the negative effects of stressful life events on physical and emotional health” [36]. The phenomenon of undergoing brachytherapy can be as concise as an instant of momentous anxiety and uncertainty [4, 5].

These patient participants fed on their internal and external assets to acclimatise to brachytherapy treatment and its side effects. This study explored gynaecological cancer patients' expectations and experiences prior to and during the brachytherapy procedure; the aim was to provide insights on how they shaped their understandings and how they mentally and emotionally processed the brachytherapy procedure as part of the cancer treatment continuum. They demonstrated very limited knowledge of their illness, but realised that they were sick and needed treatment for their illness [19]. The acknowledgement of their illness was a big step for most; they had come that far through their illness and treatment journey [37]. They had “one” more unfamiliar event in the treatment chain lying ahead of them at that stage and most participants were [37, 38], it seemed, unprepared and did not know what to expect of this upcoming HDR brachytherapy treatment. This unpreparedness and sense of uncertainty caused them to feel insecure, scared and anxious [9, 16]. In some cases, they tried to be brave and were taking some control of their circumstances by wanting to see for themselves. This action gave them hope [33, 39]. Patients did not want to hear negative stories from fellow patients, but still seemed able to influence one another; for they still talked amongst themselves [16]. Although there were many positives in conducting the study, there were also several limitations in this study. There was a limitation of only capturing the participants' voices on tape recorder and not video recording patient expressions and physical actions, for some non-verbal data might have been lost [40]. Further, when describing their pain levels, the researcher was fully aware that with the prolonged engagement between the treatment and the interview, patients might not have been experiencing pain anymore and the effect thereof lessened. The study's focus was on women and their experience of HDR intra-cavitary brachytherapy, thus no long-term side-effects or problems were taken into consideration. Different diagnoses, procedures, ages, race, education ranges and language types were included within this study; however, it was still a very small sample size. The submissive stance to the processes and procedures of the patients was not further investigated by the researcher. Further studies should aim to incorporate investigation into this finding to establish the reasons thereof.

## Conclusion

Within the South African context and geographical location, this study is one of the very few studies which explored the brachytherapy patients' expectation, experiences and understanding concerning the HDR brachytherapy procedure; the study was comprised of three stages of interviews to obtain the required data. Gynaecological cancer patients undergoing brachytherapy are faced with psychological and physical challenges [1-3, 8, 9, 16, 17]. This study identified women's unmet needs that contribute towards the development of patient care. It is envisioned that the analysis and interpretations of these patient participants” perspectives can influence the practice of health professionals in the clinical setting of brachytherapy. It is a time of individual development, where the patient participant makes decisions about her health and explores her ideals. Being informed influences how the patient participant is able to deal with the brachytherapy treatment; to get through-and to go on with her “normal” life.

### What is known about this topic

- Cancer patients are often fearful and experience anxiety, when going to the radiotherapy department because of a lack of knowledge of, and/or misconceptions about the treatment;
- It is acknowledged that being informed does not alleviate all of the patients' fears, but rather contributes to the preparation of patients on what to expect and how to cope when undergoing a radiation related treatment procedure, for instance brachytherapy;
- The interactions and communication are very important in brachytherapy as they play a significant role in the patient's experiences of the “what” and “how” of the brachytherapy as part of their treatment journey.

### What this study adds

- Patients are to some extend knowledgeable concerning their illness, but the upcoming brachytherapy treatment procedure is unfamiliar territory to them. Patient participants preferred not to be influenced by fellow patients. They wanted to experience the HDR brachytherapy treatment first hand;
- These patient participants presented with the ability to endure and tolerate the pain involved because of the hope the treatment provides them. Patients in this study described the brachytherapy as: *“cleaning the dead cells” (Pt7PostB:37), “cleaning the womb” (Pt2PreB:17-18), “some instrument inserted in my vagina” (Pt4PostB8), or “the doctor put that thing inside” (Pt8PostB:11-12)*;
- Most participants shared their desire to be healed and carry on living as normal people.

## Competing interests

The authors declare no competing interests.
